# Identification of the 3' and 5' terminal sequences of the 8 rna genome segments of european and north american genotypes of infectious salmon anemia virus (an orthomyxovirus) and evidence for quasispecies based on the non-coding sequences of transcripts

**DOI:** 10.1186/1743-422X-7-338

**Published:** 2010-11-23

**Authors:** Vikas Kulshreshtha, Molly Kibenge, Kira Salonius, Nathalie Simard, Angela Riveroll, Frederick Kibenge

**Affiliations:** 1 Department of Pathology and Microbiology, Atlantic Veterinary College, University of Prince Edward Island, Charlottetown, PEI., C1A 4P3, Canada; 2 Novartis Animal Health Canada Inc., Aqua Health Business, Victoria, PEI, C0A 2G0, Canada

## Abstract

**Background:**

Infectious salmon anemia (ISA) virus (ISAV) is a pathogen of marine-farmed Atlantic salmon (*Salmo salar*); a disease first diagnosed in Norway in 1984. This virus, which was first characterized following its isolation in cell culture in 1995, belongs to the family *Orthomyxoviridae*, genus, *Isavirus*. The *Isavirus *genome consists of eight single-stranded RNA segments of negative sense, each with one to three open reading frames flanked by 3' and 5' non-coding regions (NCRs). Although the terminal sequences of other members of the family *Orthomyxoviridae *such as *Influenzavirus A *have been extensively analyzed, those of *Isavirus *remain largely unknown, and the few reported are from different ISAV strains and on different ends of the different RNA segments. This paper describes a comprehensive analysis of the 3' and 5' end sequences of the eight RNA segments of ISAV of both European and North American genotypes, and evidence of quasispecies of ISAV based on sequence variation in the untranslated regions (UTRs) of transcripts.

**Results:**

Two different ISAV strains and two different RNA preparations were used in this study. ISAV strain ADL-PM 3205 ISAV-07 (ADL-ISAV-07) of European genotype was the source of total RNA extracted from ISAV-infected TO cells, which contained both viral mRNA and cRNA. ISAV strain NBISA01 of North American genotype was the source of vRNA extracted from purified virus. The NCRs of each segment were identified by sequencing cDNA prepared by three different methods, 5' RACE (Rapid amplification of cDNA ends), 3' RACE, and RNA ligation mediated PCR. Sequence analysis of five clones each derived from one RT-PCR product from each NCR of ISAV transcripts of segments 1 to 8 revealed significant heterogeneity among the clones of the same segment end, providing unequivocal evidence for presence of intra-segment ISAV quasispecies. Both RNA preparations (mRNA/cRNA and vRNA) yielded complementary sequence information, allowing the simultaneous identification and confirmation of the 3' and 5' NCR sequences of the 8 RNA genome segments of both genotypes of ISAV. The 3' sequences of the mRNA transcripts of ADL-ISAV-07 terminated 13-18 nucleotides from the full 3' terminus of cRNA, continuing as a poly(A) tail, which corresponded with the location of the polyadenylation signal. The lengths of the 3' and 5' NCRs of the vRNA were variable in the different genome segments, but the terminal 7 and 11 nucleotides of the 3' and 5' ends, respectively, were highly conserved among the eight genomic segments of ISAV. The first three nucleotides at the 3' end are GCU-3' (except in segment 5 with ACU-3'), whereas at the 5' end are 5'-AGU with the polyadenylation signal of 3-5 uridines 13-15 nucleotides downstream of the 5' end terminus of the vRNA. Exactly the same features were found in the respective complementary 5' and 3' end NCR sequences of the cRNA transcripts of ADL-ISAV-07, indicating that the terminal sequences of the 8 RNA genome segments are highly conserved among the two ISAV genotypes. The 5' NCR sequences of segments 1, 2, 3, 5, and 7, and the 3' NCR sequences of segments 3 and 4 cRNA were 100% identical in the two genotypes, and the 3' NCR sequences of segment 5 cRNA was the most divergent, with a sequence identity of 77.2%.

**Conclusions:**

We report for the first time, the presence of intra-segment ISAV quasispecies, based on sequence variation in the NCR sequences of transcripts. In addition, this is the first report of a comprehensive unambiguous analysis of the 3' and 5' NCR sequences of all 8 RNA genome segments from two strains of ISAV representing the two genotypes of ISAV. Because most ISAV sequences are of cDNA to mRNA, they do not contain the 3' end sequences, which are removed during polyadenylation of the mRNA transcripts. We report for the first time the ISAV consensus sequence CA^T^/_A_TTTTTACT-3' (in the message sense 5'-3') in all segments of both ISAV genotypes.

## Background

Infectious salmon anemia (ISA) virus (ISAV) is a pathogen of marine-farmed Atlantic salmon (*Salmo salar*); a disease first diagnosed in Norway in 1984 [1]. It has continued to cause major disease outbreaks in marine fish [2,3] with the clinical signs of severe anaemia, congestion of the liver and spleen along with haemorrhagic liver necrosis [4]. ISA is an OIE [Office International des Epizooties] listed disease [1]. The ISA virus was first propagated in cell culture in 1995 [5], which allowed its molecular characterization [6] and subsequent taxonomic classification to the family *Orthomyxoviridae*, genus, *Isavirus *[7].

The ISAV particles are enveloped (90-140 nm in diameter) and contain a genome of eight single-stranded (ss)RNA segments of negative polarity [1,6]. Like in other orthomyxoviruses such as influenza A virus, each RNA segment contains one to three open reading frames (ORFs) flanked by the 5' and 3' non-coding regions (NCRs). In influenza A virus, the first 12 nucleotides at the 3' end and the first 13 nucleotides at the 5' end of NCR in all the viral RNA segments are highly conserved [8-13]. These partially complementary termini base pair to form terminal panhandle structures [14], which function as promoters by interacting with the viral polymerase complex during replication and transcription of viral RNA [8,15-20]. Moreover, the segment specific NCR sequences may play important roles in virus virulence [21], and in the rescue of influenza virus using the reverse genetics system [8,22]. Although the terminal sequences of members of the family *Orthomyxoviridae *such as *Influenzavirus A *have been analyzed extensively and subsequently used in engineering recombinant viruses, those of *Isavirus *remain largely unknown, and the few reported are from different ISAV strains and on different ends of the different RNA segments [23-30]. Moreover, others have reported unique 5' terminal sequences for ISAV RNA segments 2 [31], 3 [26], 5 [27], and 8 [7], indicating a variation in the RNA templates used. Furthermore, the focussed sequencing of cDNA to ISAV mRNA to date has meant that all 3' sequences of ISAV found in the GenBank Database [32] till now (except for genome segments 6 [27,28] and 7 [23,33]) are incomplete since the 3' terminal sequences downstream of the orthomyxoviral polyadenylation signal are removed during polyadnyelation [34].

Sequence analysis of several ISAV isolates in the ORFs on the eight genomic RNA segments consistently reveals two genotypes that are designated with respect to their geographic origin, European and North American; the two show 15-19% difference in their amino acid sequences of the surface glycoproteins, fusion (F) protein and haemagglutinin-esterase (HE) protein [35]. It has been proposed to designate the European genotype as Genotype I and the North American genotype as Genotype II because the virus has now been reported in Europe, North America, and South America [3]. ISAV isolates can be further differentiated on the basis of insertion/deletions in a highly polymorphic region (HPR) spanning residues ^337^V to M^372 ^in the stem of the HE protein, adjacent to the transmembrane region [36], but the HPR is vaguely defined [37], and has been rejected in epidemiological investigations because HPR groups vary significantly and are not suited as an indicator of relatedness between virus isolates [38]. There is a lack of information on the genetic changes in NCRs of any RNA segment of ISAV although these sequences are known to play a vital role in replication of orthomyxoviruses [8,15].

**Figure 1:**
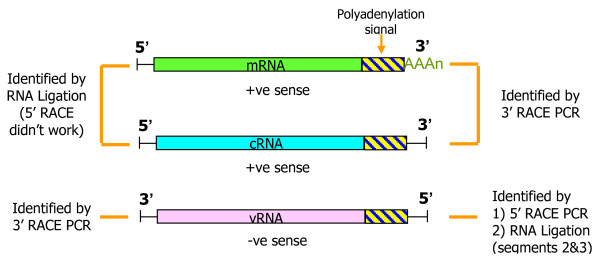
**Experimental design of the study**. Diagram illustrating the different types of template ISAV RNA used and the method of determining the non-coding regions sequences. The mRNA is shown polyadenylated. Not drawn on scale.

ISAV being an orthomyxovirus is characterized by abundant genetic variation. Orthomyxoviruses such as influenza viruses have high mutation rates because the viral RNA-dependent RNA polymerases have a high misincorporation frequency and have no proofreading-repair mechanisms [39]. Moreover, mismatch repair mechanisms are unlikely to operate on replicating RNA [40] and cannot operate on ssRNA progeny genomes [39]. In addition, RNA viruses generally have very short replication times and generate very high virus yields [41], two characteristics that strongly accelerate evolution of RNA viruses. As a consequence, viral quasispecies populations [39,40] are present wherever orthomyxoviruses multiply in a host [42,43], and are connected with a high potential for rapid evolution [44,45] since the multiple variants are subjected to continuous adaptation pressure [46]. Quasispecies in influenza A virus isolates and samples have been readily identified using the highly discriminating methodologies of Mass Spectrometry coupled to RT-PCR [47], and high-resolution genome sequencing [43]. There is very limited information on the existence of ISAV quasispecies populations. In the report on ISAV terminal sequences of segments 7 and 8 [23], the mRNA transcripts of these segments showed heterogeneous 5' ends suggestive of a quasispecies, however this was not further pursued as the heterogeneity was attributed only to cap-stealing that is characteristic of influenza virus mRNA synthesis [24,48]. Kibenge *et al*. [3] used sequence analysis of RT-PCR products of ISAV segment 6 ORF obtained directly from fish tissue and found 24 distinct HPR variants associated with the 2007-2009 ISA epizootic in Chile, but only 7 distinct ISAV strains based on segments 5 and 6 phylogenetic analyses. The appearance of multiple HPR groups in such a short time in tissues from the same or different fish originating from the same or different fish farms indicated that the ISAV HPR groups existed as quasispecies populations [3]. To examine further the genetic diversity of ISAV transcripts and the intra-segment viral evolution of ISAV, the terminal sequences of an RT-PCR product from each end of the eight RNA segments of ISAV strain ADL-ISAV-07 were determined. Because of the vital role they play in virus replication in orthomyxoviruses [9], it was considered that the diversity of terminal sequences in a population of viruses present in an infected cell lysate as represented by RT-PCR would be a true indication of ISAV quasispecies. In addition, the 3' and 5' NCR sequences of each of the eight segments of the ISAV genome were determined from vRNA extracted from purified virus particles of ISAV strain NBISA01. The two different ISAV strains belong to the two genotypes of ISAV (ADL-ISAV-07 is of European genotype and NBISA01 is of North American genotype) and each was a source of a different RNA type (mRNA/cRNA versus vRNA) such that the NCR sequences obtained were complementary, allowing the simultaneous identification and confirmation of the 3' and 5' NCR sequences of the 8 RNA genome segments of both genotypes of ISAV. The experimental design of this study is illustrated in Figure 1.

## Results

### Non-coding sequences of ISAV RNA segments determined from transcripts of ISAV strain ADL-ISAV-07

**Figure 2:**
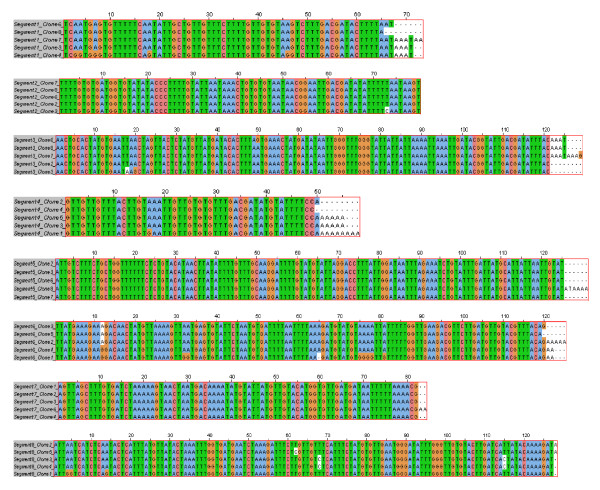
**Alignment of cDNA sequences of 3' UTRs of mRNA transcripts of segments 1 to 8 of ISAV strain ADL-ISAV-07**. Predicted 3' NCR sequences are of five different clones analyzed for each genome segment of ISAV using the 3' RACE method; S1 = RNA segment 1; S2 = RNA segment 2; S3 = RNA segment 3; S4 = RNA segment 4; S5 = RNA segment 5; S6 = RNA segment 6; S7 = RNA segment 7; S8 = RNA segment 8. The dash (-) denotes deletion.

The 5' and 3' UTR sequences of viral RNA segments were determined using the Rapid Amplification of cDNA ends (RACE) method. For performing the 3' RACE, total RNA containing viral mRNA and cRNA was reverse transcribed to cDNA, which was then amplified by PCR using the ISAV segment specific primers (Table 1) and adapter primer (Invitrogen). The adapter primer has 17-dT residues and an adapter sequence with three restriction endonuclease sites. Since the long stretches of dT residues do not base pair well, the specificity of amplification was improved by performing a second PCR using the ISAV segment specific primers (Table 1) and the universal amplification primer (AUP). The nucleotide sequences of the cDNA of 3' UTRs of five clones of each ISAV segment transcript are shown in Figure 2, with the 17-dA residues of the poly-A tail removed.

**Table 1:** Reverse primers1 used in 3 'RACE for amplification of 3' non-coding region of different ISAV segments using mRNA transcripts as template

ISAV RNA segment	Primer sequence
**Segment 1**	ssp3: 5'-CTGCTATGGAGAGGTGTATG-3'

**Segment 2**	ssp3: 5'-GACTTGGACCCTCAACAGATC-3'

**Segment 3**	ssp3: 5'-GCTAGAACATGTGTAGCTGTG-3'

**Segment 4**	ssp3: 5'-TGGCAACATGACCCTGAGCT-3'

**Segment 5**	ssp3: 5'-GAGTTCATCAAATGCTGTGGA-3'

**Segment 6**	ssp3: 5'-GTTCCTCACTGATGCATTGAC-3'

**Segment 7**	ssp3: 5'-GATGGTCTGTCACACATGC-3'

**Segment 8**	ssp3: 5'-CTCTCTACTGTGTGATGAAAGA-3'

^1 ^The forward primer for PCR was the Universal Amplification Primer (UAP) that comes with the 3'RACE kit (Invitrogen).

**Figure 3:**
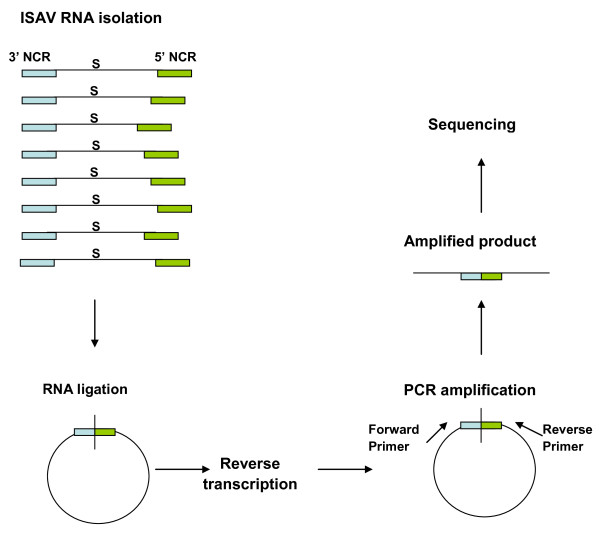
**A diagram depicting RNA ligation strategy used for ligating ISAV RNA**. Total RNA was isolated from ISAV-infected cell lysates and was ligated using RNA Ligase. A cDNA was synthesized from ligated RNA by Reverse Transcriptase using the random primers. PCR was performed on the cDNA template using segment specific primers. The amplified product was gel-purified, cloned in a plasmid and plasmid DNA sequencing was performed.

**Figure 4:**
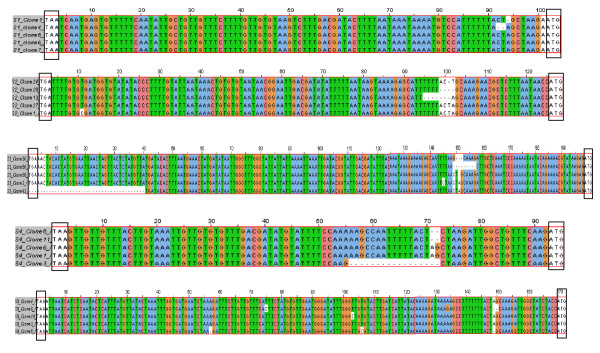
**Alignment of cDNA sequences of 5' and 3' UTRs of cRNA transcripts of segments 1 to 4 of ISAV strain ADL-ISAV-07**. Predicted 5' and 3' UTR sequences are of five different clones analyzed for each genome segment of ISAV using the RNA ligation strategy; S1 = RNA segment 1; S2 = RNA segment 2; S3 = RNA segment 3; S4 = RNA segment 4. The dash (-) denotes deletion. The arrow indicates the junction of 3' and 5' UTRs of the cRNA transcript for each segment. The stop codons (TAA, TGA) and start codon (ATG) of each open reading frame are enclosed in a box at the ends of the alignments.

**Figure 5:**
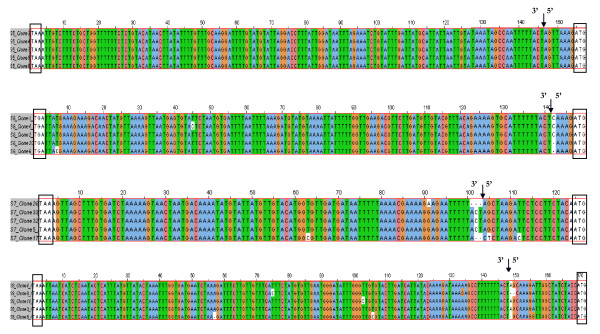
**Alignment of cDNA sequences of 5' and 3' UTRs of cRNA transcripts of segments 5 to 8 of ISAV strain ADL-ISAV-07**. Predicted 5' and 3' UTR sequences are of five different clones analyzed for each genome segment of ISAV using the RNA ligation strategy; S5 = RNA segment 5; S6 = RNA segment 6; S7 = RNA segment 7; S8 = RNA segment 8. The dash (-) denotes deletion. The arrow indicates the junction of 3' and 5' UTRs of the cRNA transcript for each segment. The stop codons (TAA, TGA) and start codon (ATG) of each open reading frame are enclosed in a box at the ends of the alignments.

The 5' RACE protocol with ISAV segment specific primers (Table 2) did not work for total RNA for any ISAV segment despite repeated attempts. An RNA ligation method as depicted in Figure 3[8,49] was therefore used to obtain both the 5' and 3' end sequences. For this, the 5' and 3' ends of the ISAV transcripts in total RNA were ligated using T4 RNA Ligase to form a single stranded RNA circle. Using the appropriate primers (Table 3), the circular RNA spanning the ligation junction was reverse transcribed and amplified by PCR. These PCR products were cloned using the TOPO TA cloning kit (Invitrogen) followed by plasmid DNA sequencing. The nucleotide sequences of the cDNA of the complete 5' and 3' UTRs of five clones of each ISAV segment transcript, from the stop codon (TAA or TGA) to the first codon (ATG) of the ORF, are shown in Figures 4 and 5. The absence of a poly(A) tail in these sequences for all eight ISAV segments indicated that only viral cRNA molecules (Figure 1) had been circularized, and that we had obtained the full 5' termini and full 3' termini of all eight ISAV segments of ISAV strain ADL-ISAV-07 including any accompanying genetic variation within each segment.

**Table 2:** Reverse primers1 used in 5'RACE for amplification of 5' non-coding region of different ISAV segments using mRNA transcripts as template

ISAV RNA segment	Primer sequence
**Segment 1**	ssp1: 5'-GCTCCAACTGCTGCTCTAC-3'
	
	ssp2: 5'-CAGTACTCCTCGGTAGTTGG-3'

**Segment 2**	ssp1: 5'-GTGTTCAATCAGGTCTGACAT-3'
	
	ssp2: 5'-CTCTGAAACCTGTTGCTACTAT-3'

**Segment 3**	ssp1: 5'-GTAACATCTGCCATGTCCAC-3'
	
	ssp2: 5'-CTTCTGACAGGATTGCGCTG-3'

**Segment 4**	ssp1: 5'-CGTATGGCCCTCCTCTGTAA-3'
	
	ssp2: 5'-CATGTGCTGATCGGTGTTGG-3'

**Segment 5**	ssp1: 5'-GTCTGGTACAGAATGGAACG-3'
	
	ssp2: 5'-GTGCAACAGCTATCCAAGTC-3'

**Segment 6**	ssp1: 5'-GCGTCTGCTCGTCCAACAAGT-3'
	
	ssp2: 5'-GCGTTGTCCAGTGTCATCGA-3'

**Segment 7**	ssp1: 5'-CAGACACTCCTCATAGTACC-3'
	
	ssp2: 5'-GCATGTGTGACAGACCATC-3'

**Segment 8**	ssp1: 5'-CATCTGCATCCTGCTGTGTAG-3'
	
	ssp2: 5'-CTTTCATCACACAGTAGAGAG-3'

^1 ^The forward primer for PCR in all cases (i.e., with either ssp1 or ssp2) was the Universal Amplification Primer (UAP) that comes with the 5'RACE kit (Invitrogen).

**Table 3:** Primers used in amplification of the non-coding region of different ISAV segments using cDNA from ligated mRNA transcripts as template

ISAV RNA segment	Primer sequence
**Segment 1**	Forward: 5'-CTGATGGATGAATATGGTGT-3'
	
	Reverse: 5'-GCTCCAACTGCTGCTCTAC-3'

**Segment 2**	Forward: 5'-CTTATCGACGAGGTGGAGGT-3'
	
	Reverse: 5'-CTCTGAAACCTGTTGCTACTAT-3'

**Segment 3**	Forward: 5'-GAGTTCGATGAGGACGATGA-3'
	
	Reverse: 5'-CTTCTGACAGGATTGCGCTG-3'

**Segment 4**	Forward: 5'-CAGAAAATTGCAGTCAGTACC-3'
	
	Reverse: 5'-CATGTGCTGATCGGTGTTGG-3'

**Segment 5**	Forward: 5'-GCTGATTGTAGTTGTGTTGGT-3'
	
	Reverse: 5'-GTCTGGTACAGAATGGAACG-3'

**Segment 6**	Forward: 5'-CATGTACAAGTCTAGAGGTAG-3'
	
	Reverse: 5'-GCGTTGTCCAGTGTCATCGA-3'

**Segment 7**	Forward: 5'-GCTTAGGGCTGGACTTCACT-3'
	
	Reverse: 5'-GCATGTGTGACAGACCATC-3'

**Segment 8**	Forward: 5'-GCATCCTGCTCGACAGAGAA-3'
	
	Reverse: 5'-CTTTCATCACACAGTAGAGAG-3'

### ISAV quasispecies based on non-coding terminal sequences

In designing this study, it was considered that the diversity of terminal sequences in a population of viruses present in an infected cell lysate as represented by RT-PCR would be a true indication of ISAV quasispecies. For this, two types of viral transcripts were studied; viral mRNA transcripts were analyzed using the 3'RACE protocol whereas viral cRNA transcripts were analyzed using RNA ligation method (Figure 1). Thus, a large number of clones of cDNA to 3' UTRs of viral mRNA transcripts and to 5' and 3' UTRs of viral cRNA transcripts of segments 1 to 8 were screened and sequenced, and the first five clones with good sequences in each case were used for further analysis.

The lengths of the 3' UTR sequences for mRNA transcripts of the different segments (Figure 2), and the 5' and 3' NCR sequences for cRNA of the different segments (Figures 4 and 5) were variable. However, significant homology was observed among the five different clones of each end of the same segment in both viral mRNA and cRNA. Interestingly, mutations and/or deletions were observed in both the 3' UTR sequences of mRNA and 5' and 3' NCR sequences of cRNA of all the segments except in the NCRs of segment 5 cRNA, which were highly conserved. Deletions were more prevalent in clones of the viral cRNA (Figures 4 and 5) whereas nucleotide substitutions were mainly observed in the viral mRNA (Figure 2) of the same segment. The majority of the mutations in the cDNA to viral mRNA were A→G or T→C mutations. The presence of these mutations and deletions in clones of the same RNA segment were considered to indicate intra-segment ISAV quasispecies. In order to document the quasispecies of each NCR of ISAV RNA segment, a heterogeneity index (HI) [50] was calculated as the proportion of ISAV clones for a particular terminus of an RNA segment not bearing the predominant sequences. All 3' UTR sequences of mRNA were heterogenous, with segments 2, 5, and 7 having the lowest HI (0.2) and segments 1, 3, and 6 having the highest HI (0.8) (Table 4). Of all ISAV NCR sequences, the lowest HI (0.0) was found in the 5' and 3' NCRs of segment 5 cRNA in which all five clones had identical sequence (Table 5). The 5' and 3' NCRs of segment 1 cRNA, and the 3' NCR of segment 4 cRNA also had low HI (0.2) since four of the five clones in each case were identical. The 3' NCR of segment 3 cRNA had the highest HI (0.8) of all NCRs of ISAV cRNA, with all five clones having different sequences (Table 5).

**Table 4:** Heterogeneity of ISAV 3' untranslated regions in viral mRNA1

ISAV RNA segment	Number of clones	Number of different sequences	Heterogeneity Index (HI)	Frequency of sequences
**Segment 1**	5	5	0.8	1 (1), 1(1), 1(1), 1(1), 1(1)

**Segment 2**	5	2	0.2	1 (1), 1(4)

**Segment 3**	5	5	0.8	1 (1), 1(1), 1(1), 1(1), 1(1)

**Segment 4**	5	4	0.6	1 (1), 1(1), 1(1), 1(2)

**Segment 5**	5	2	0.2	1 (1), 1(4)

**Segment 6**	5	5	0.8	1 (1), 1(1), 1(1), 1(1), 1(1)

**Segment 7**	5	2	0.2	1 (1), 1(4)

**Segment 8**	5	4	0.6	1 (1), 1(1), 1(1), 1(2)

^1^Sequence obtained using the 3' RACE method.

**Table 5:** Heterogeneity of ISAV 5' and 3' non-coding regions in viral cRNA1

Non-coding region of ISAV RNA segment	Number of clones	Number of different sequences	Heterogeneity Index (HI)	Frequency of sequences
**5' end Segment 1**	5	2	0.2	1 (1), 1(4)
		
**3' end Segment 1**		2	0.2	1 (1), 1(4)

**5' end Segment 2**	5	2	0.6	1 (2), 1(3)
		
**3' end Segment 2**		4	0.6	1 (1), 1(1), 1(1), 1(2)

**5' end Segment 3**	5	3	0.4	1 (1), 1(1), 1(3)
		
**3' end Segment 3**		5	0.8	1 (1), 1(1), 1(1), 1(1), 1(1)

**5' end Segment 4**	5	2	0.6	1 (2), 1(3)
		
**3' end Segment 4**		2	0.2	1 (1), 1(4)

**5' end Segment 5**	5	1	0.0	1 (5)
		
**3' end Segment 5**		1	0.0	1 (5)

**5' end Segment 6**	5	2	0.6	1 (2), 1(3)
		
**3' end Segment 6**		3	0.4	1 (1), 1(1), 1(3)

**5' end Segment 7**	5	2	0.6	1 (2), 1(3)
		
**3' end Segment 7**		3	0.4	1 (1), 1(1), 1(3)

**5' end Segment 8**	5	3	0.4	1 (1), 1(1), 1(3)
		
**3' end Segment 8**		4	0.6	1 (1), 1(1), 1(1), 1(2)

^1^Sequence obtained using the RNA ligation method.

Heterogeneity index (HI) is calculated as: 1-(proportion of clones bearing majority of the sequence). The number preceding the parentheses indicates a sequence while the number within the parentheses indicates the numbers of clones in which that sequence appear.

### Non-coding sequences of RNA segments of ISAV determined from vRNA of NBISA01

The 3' and 5' end sequences of the NCRs of RNA segments of ISAV were confirmed from RNA extracted from purified virus, i.e., vRNA, using 3' and 5' RACE, respectively, and appropriate primers (Tables 6 and 7). For ISAV segments 2 and 3 vRNA, the 5' RACE did not work. In this case, the RNA ligation method (Figure 3) [8,49] was used without tobacco acid pyrophosphatase (TAP) treatment for segment 2 and with TAP treatment for segment 3, with the appropriate primers (Table 8); PCR was performed on the cDNA spanning the ligation junction.

**Table 6:** Reverse primers1 used in 3'RACE for amplification of 3' non-coding region of different ISAV segments using vRNA as template

ISAV RNA segment	Primer sequence
**Segment 1**	ssp4: 5'-GCTGGTTCTGTTGAAGGACAT-3'

**Segment 2**	ssp4: 5'-GTCAGCCACGCCTACAGTT-3'

**Segment 3**	ssp4: 5'-CTTCATCATCTACACCAGCCA-3'

**Segment 4**	ssp4: 5'-CATGTCTAGCACTCCGTGTG-3'

**Segment 5**	ssp4: 5'-CACTGCACAGTTCTCCATCT-3'

**Segment 6**	ssp4: 5'-GTCGGTATGATCATGTCGTTA-3'

**Segment 7**	ssp4: 5'-CATACGACCAGATCATCACTG-3'

**Segment 8**	ssp4: 5'-CATCGTCGCTCGGCTGATC-3'

^1^The forward primer for PCR was the Universal Amplification Primer (UAP) that comes with the 3'RACE kit (Invitrogen).

**Table 7:** Reverse primers1 used in 5'RACE for amplification of 5' non-coding region of different ISAV segments using vRNA as template

ISAV RNA segment	Primer sequence
**Segment 1**	ssp1: 5'-GTAGTGGCAAGTGCTAGATAC-3'
	
	ssp2: 5'-GCCATGAGGTGCTGCATTG-3'

**Segment 2**	ssp1: 5'-GAAGCTGTGAACAGAGGACAT-3'
	
	ssp2: 5'-GCTGCAGCATAGAGTTCGAT-3'

**Segment 3**	ssp1: 5'-GGTACAGATCCAACATCAAG-3'
	
	ssp2: 5'-GCAAGTGAACGTGACTGACA-3'

**Segment 4**	ssp1: 5'-CTCTGTGCAAACAAAAGCAGA-3'
	
	ssp2: 5'-TGGCAACATGACCCTGAGCT-3'

**Segment 5**	ssp1: 5'-GAGGAGGATTGCCTATATGAAC-3'
	
	ssp2: 5'-CAAGTGGAAATCGGACTAATAG-3'

**Segment 6**	ssp1: 5'-GATGAGTACGTTGACACAC-3'
	
	ssp2: 5'-CAGAGGCACTGACATGTCCA-3'

**Segment 7**	ssp1: 5'-CACTTGCCCAGACGAGTAC-3'
	
	ssp2: 5'-CAGTGACAAGGACGAGATGC-3'

**Segment 8**	ssp1: 5'-GATCAGTCGAGCGACGATGA-3'
	
	ssp2: 5'-GCATCCTGCTCGACAGAGAA-3'

^1^The forward primer for PCR in all cases (i.e., with either ssp1 or ssp2) was the Universal Amplification Primer (UAP) that comes with the 5'RACE kit (Invitrogen).

**Table 8:** Primers used in amplification of the non-coding region of different ISAV segments using cDNA from ligated vRNA as template

ISAV RNA segment	Primer sequence
**Segment 2**	Forward: 5'-GTCAGCCACGCCTACAGTT-3'
	
	Reverse: 5'-GCTGCAGCATAGAGTTCGAT-3'

**Segment 3**	Forward: 5'-CTTCATCATCTACACCAGCCA-3'
	
	Reverse: 5'-GCAAGTGAACGTGACTGACA-3'

#### 3' end of vRNA

**Figure 6:**
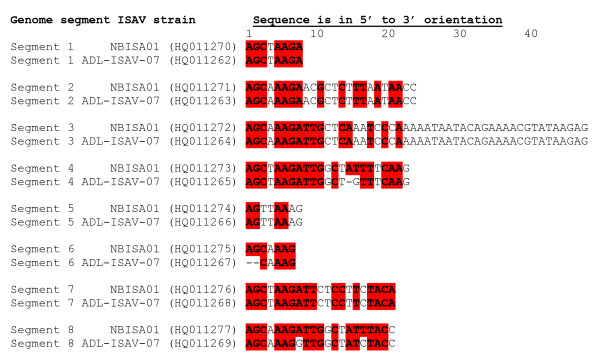
**Alignment of cDNA sequences of the complete 5' non-coding regions of cRNA of the different genomic segments of ISAV strains ADL-ISAV-07 and NBISA01 of European and North American genotypes, respectively**. The GenBank Accession numbers are given in brackets. Only the non-coding sequences are shown up to the start codon (not included) of the open reading frame. Genome segment sequences were aligned manually. Sequences conserved between all genome segments are in bold red. Note the variation in length between different genome segments of the same virus.

The length of the 3' NCR in the different genome segments was variable, ranging from seven nucleotides in segment 6 to 48 nucleotides in segment 3 (Figure 6). The terminal 7 nucleotides were conserved in all eight segments except for segment 5 at position 3 with a C→T mutation, segments 1, 4, 5, and 7 at position 4 with A→T mutation, and segment 5 at position 7 with a G→A mutation. Exactly the same features were found in the respective complementary 5' end sequences of the cRNA transcripts of ADL-ISAV-07 (Figures 4 and 5). The first three nucleotides at the 3' end in all members of *Orthomyxoviridae *are GCU-3' (except in ISAV segment 5 with ACU-3'), and the length of the non-coding region in orthomyxoviruses other than ISAV is 20-23 nucleotides long [51-54]. Table 9 summarises the structural features of the NCRs of both genotypes of ISAV. The 5' NCR sequences of segments 1, 2, 3, 5, and 7 were 100% identical in the two genotypes of ISAV. The 5' NCR sequences of segment 6 were also identical in the two genotypes except that ISAV strain ADL-ISAV-07 had a deletion of the first 2 nucleotides, which was present in all 5 clones that were analyzed.

**Table 9:** Comparison of the lengths of the non-coding regions of ISAV genomic segments between the two ISAV genotypes (see also Figures 6 and 7)

ISAV RNA segment cRNA	5' Non-coding region			**3' Non-coding region**^**1**^		
	**NBISA01**^**2**^	**ADL-ISAV-07**	**Percent identity**	**NBISA01**^**2**^	**ADL-ISAV-07**	**Percent identity**

**Segment 1**	8	8	100	89 (15-4)	90* (15-4)	86.5

**Segment 2**	24	24	100	98 (14-4)	98 (14-4)	99.0

**Segment 3**	48	48	100	147 (14-4)	147 (14-4)	100

**Segment 4**	23	22*	91.3	67 (13-5)	67 (13-5)	100

**Segment 5**	8	8	100	143 (15-3)	143 (15-3)	77.2

**Segment 6**	7	5*	Not done	131 (14-4)	138* (14-4)	80.0

**Segment 7**	21	21	100	99 (14-4)	100* (14-4)	87.0

**Segment 8**	21	21	90.5	145 (13-5)	144* (13-5)	89.0

^1 ^Numbers in brackets denote # nucleotides to polyadenylation signal, and length of polyadenylation signal.

^2^Lengths of non-coding regions deduced from vRNA sequences.

*Deletions/insertions revealed by sequence alignment.

#### 5' end of vRNA

**Figure 7:**
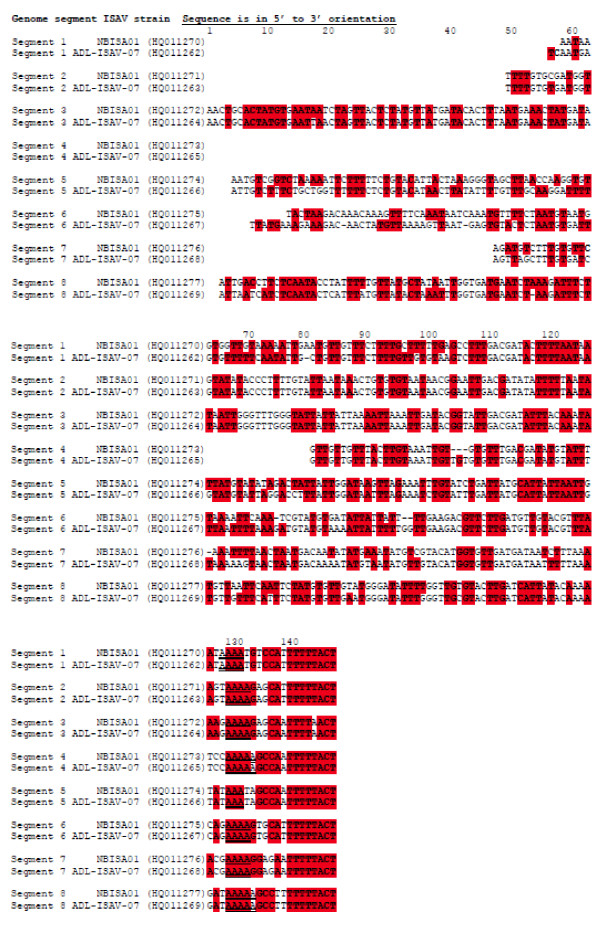
**Alignment of cDNA sequences of the complete 3' non-coding regions of cRNA of the different genomic segments of ISAV strains ADL-ISAV-07 and NBISA01 of European and North American genotypes, respectively**. The GenBank Accession numbers are given in brackets. Only the non-coding sequences are shown, starting from the stop codon (not included) of the open reading frame. Genome segment sequences were aligned manually. Sequences conserved between all genome segments are in bold red. The polyadenylation signal sequences are double underlined. Note the variation in length between different genome segments of the same virus. The nucleotide positions are relative to the longest 3' NCR, which is found in segment 3.

The length of the 5' NCR in all eight ISAV segments was significantly longer than the corresponding 3' NCR (Figure 7). It was also variable in the different genome segments, ranging from 67 nucleotides in segment 4 to 147 nucleotides in segment 3 (Figure 7; Table 9). The 5' terminal 11 nucleotides were conserved in all eight segments except for segment 3 at position 4 with a T→A mutation, segments 3, 4, 5, and 7 at position 9 with T→A mutation, segment 8 at position 10 with A→T mutation, and segment 7 at position 11 with a C→G mutation. Exactly the same features were found in the respective complementary 3' end sequences of the cRNA transcripts of ADL-ISAV-07 (Figures 4 and 5). The first three nucleotides at the 5' end of vRNA are 5'-AGU (or 5'-TCA in the cDNA), with the polyadenylation signal 13-15 nucleotides downstream of the 5' end terminus of the vRNA. This is also true for other members of *Orthomyxoviridae *[8,15]. In both NBISA01 vRNA (Figure 7) and ADL-ISAV-07 (Figures 4 and 5), the polyadenylation signal sequence was identical in each genome segment although the length varied between 3 and 5 uridines, depending of the segment. ISAV RNA segment 5 had the shortest, 3 uridines, while segments 4 and 8 had the longest, 5 uridines and the rest of the segments had 4 uridines. As summarized in Table 9, the 3' NCR sequences of segments 3 and 4 cRNA were 100% identical in the two genotypes of ISAV, whereas the 3' NCR sequence of segment 5 cRNA was the most divergent, with a sequence identity of 77.2%.

#### Secondary structures of terminal sequences

**Figure 8:**
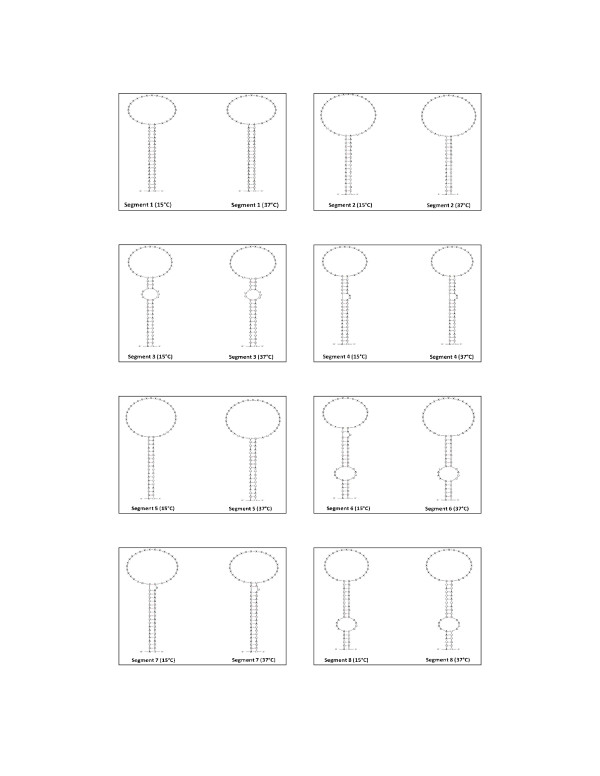
**Predicted secondary structure of 5' and 3' terminal sequences of vRNA of ISAV strain NBISA01 at 15°C and 37°C**. The letter X indicates the segment specific sequences.

**Figure 9:**
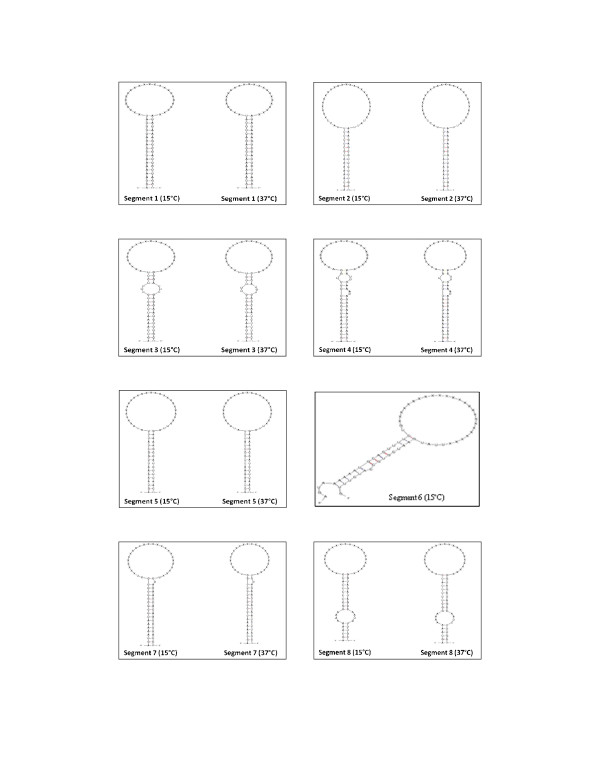
**Predicted secondary structure of 5' and 3' terminal sequences of vRNA of ISAV strain ADL-ISAV-07 at 15°C and 37°C**. Note that there is no prediction of segment 6 structure of ADL-ISAV-07 at 37°C since the nucleotides A and G are missing from 3' end of this sequence. The letter X indicates the segment specific sequences.

The sequences of 3' and 5' terminal sequences of genomic segments of orthomyxovirus are partially complementary, which results in the formation of double helical structures known as panhandle structures [55]. The predicted secondary structures of terminal sequences of the eight segments of ISAV strains NBISA01 and ADL-ISAV-07 at 15°C and 37°C are shown in Figures 8 and 9. Those of the NCRs of influenza A viruses are reported to be 21-24 nucleotides of which the self-complementary 3' and 5 termini consist of terminal 12-13 nucleotides [24]. The present study shows that only 7-11 terminal nucleotides are conserved in both genotypes of ISAV, resulting in slightly fewer self-complementary nucleotides in the secondary structures of genomic segments of ISAV.

## Discussion

The present study focussed on the quasispecies distribution found in the NCRs of mRNA/cRNA of each genome segment of ISAV. Comparison of 3' UTR sequences of mRNA transcripts from the different segments revealed heterogeneity among the five different clones of the same segment for all RNA segments. The 5' and 3' NCRs of the cRNA from the different segments were also variable; the NCRs of segment 5 cRNA were the least variable with all five clones showing identical sequence, whereas the 3' NCR of segment 3 cRNA had the highest HI (0.8) of all NCRs of ISAV cRNA, with all five clones having different sequences (Table 5). In a previous report on ISAV terminal sequences of segments 7 and 8 [23], two different sets of sequences, with and without 5'-end heterogeneous extensions were found; the heterogeneity was attributed to cap-stealing that is characteristic of influenza virus mRNA synthesis [48], whereas the sequences without heterogenous extensions were attributed to viral cRNA. In the present study, we were not able to obtain any sequences from the 5' ends of viral mRNA because our 5' RACE protocol did not work with the total RNA preparations, and the RNA ligations worked only for viral cRNA. Therefore, the heterogeneity demonstrated in the present study cannot be explained by cap-stealing, as it consisted mostly of deletions in the 5' NCR sequences of the cRNA (Figures 4 and 5). Moreover, the heterogeneity was also found in the 3' NCR sequences of cRNA (Figures 4 and 5) and in 3' UTR sequences of viral mRNA (Figure 2). It is our considered opinion that this sequence variation in terminal sequences of the same segment end is suggestive of intra-segment ISAV quasispecies. It is interesting that on one hand both 5' and 3' NCRs of segment 5 (F gene) cRNA showed no variation in the five clones while on the other extreme all five clones in each of the 3' NCR of segment 3 (NP gene) cRNA, and the 3' UTR sequences of mRNA of segments 1, 3, and 6 (PB1, NP, and HE genes, respectively) had different sequences.

The biological significance of quasispecies in the terminal NCR sequences of ISAV is not known at this time. We speculate that the quasispecies detected may play an important role in ISAV replication. For replication in influenza virus, it is known that viral RNA dependent RNA polymerase initiates the RNA synthesis on viral RNA by binding to the panhandle structure formed as a result of partial complementarity of 3' and 5' non-coding sequences of viral RNA [23]. Therefore, any alteration in the sequence of non-coding region may affect the complementarity of 3' and 5' non-coding sequences, thereby affecting the formation of panhandle structure and viral replication as evidenced in the present study in which the deletion of nucleotides (A and G) from the 3' non-coding region of segment 6 (of ADL-ISAV-07) affected the formation of panhandle structure (Figure 9). Moreover, quasispecies may also play a role at the level of protein expression. Wang and Lee [10] observed an alteration in the protein expression level as a result of induction of mutation in the non-coding regions of PB1 and PA genes of Influenza A virus. On the basis of that study, it is possible that any change in the sequence of non-coding regions in ISAV may affect protein expression as well. The transcripts of ISAV were analyzed at 15°C since this is the optimal growth temperature for ISAV. The transcription and replication of ISAV is based on the influenza virus model system and the predicted secondary structures of ISAV segments appear to be analogous to the panhandle structures for influenza virus. Therefore, the stability of ISAV panhandle structures at 15°C was compared to that of the influenza virus panhandle formation at 37°C (Figures 8 and 9).

We report for the first time a comprehensive unambiguous analysis of NCR sequences of all eight RNA genome segments from two strains of ISAV belonging to the two ISAV genotypes. The experimental design used (Figure1), whereby the viral mRNA/cRNA was from ISAV strain ADL-ISAV-07 of European genotype and the vRNA was from ISAV strain NBISA01 of North American genotype, such that the NCR sequences obtained were complementary, allowed the simultaneous identification and confirmation of the 3' and 5' NCR sequences of the 8 RNA genome segments of both ISAV genotypes. The terminal sequences of the 8 RNA genome segments are highly conserved among the two ISAV genotypes. The 3' NCRs of each ISAV RNA gene segment are of variable lengths and the orthomyxoviral consensus sequence 5'-AGCAAAGA (in the message sense 5'-3') is present in all segments except for segment 5 at position 3 with a C→T mutation, segments 1, 4, 5, and 7 at position 4 with A→T mutation, and segment 5 at position 7 with a G→A mutation. Therefore, consistent with other reports of the ISAV 5' NCR sequences [25-30], we have confirmed the identity of the ISAV consensus 5' end sequences (Figure 6). Therefore, the first three nucleotides at the 3' end of vRNA in all members of *Orthomyxoviridae *are GCU-3' (except in ISAV segment 5 with ACU-3').

**Figure 10:**
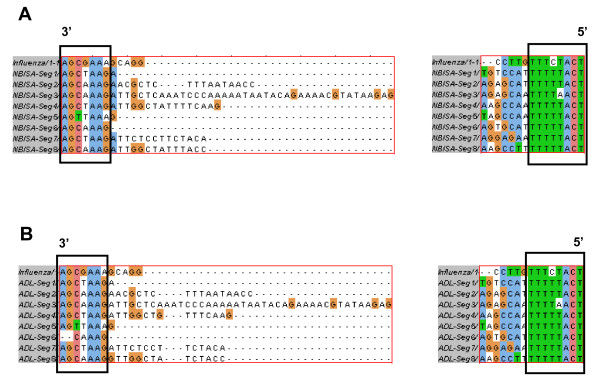
**Alignment of influenza promoter sequences with the non-coding sequences of ISAV (ADL strain ADL-ISAV07 and NBISA strain NBISA01)**. (**A**) Alignment of 3' and 5' promoter sequences of influenza with 3' and 5' non coding regions of ISAV strain NBISA01. Sequences in the closed box predict the promoter sequences of NBISA01. (**B**) Alignment of 3' and 5' promoter sequences of influenza with the consensus sequences of 3' and 5' non coding regions of ISAV strain ADL-ISAV-07. Sequences in the closed box predict the promoter sequences of ADL-ISAV-07.

However, a BLAST search [56] of the GenBank Database [32] revealed additional unique 5' terminal sequences present on ISAV RNA segments 2 [31], 3 [26], 5 [27], and 8 [57,58]. These sequences, which range from 1 to 12 additional nucleotides at the 5' ends (Table 10), are probably due to sequencing mRNA or circularized cRNA templates resulting in appearance of heterogenous sequences at the 5' ends. The 5' NCRs in the present study were also of variable lengths on the different RNA segments, and were also characterized by an orthomyxoviral consensus sequence CA^T^/_A_TTTTTACT-3' (in the message sense 5'-3') in all segments except for segment 3 at position 4 with a T→A mutation, segments 3, 4, 5, and 7 at position 9 with T→A mutation, segment 8 at position 10 with T→A mutation, and segment 7 at position 11 with a C→G mutation. A BLAST search [56] of the GenBank Database [32] revealed that all ISAV 3' terminal sequences reported to date are incomplete (i.e., they lack the consensus sequence) except for genome segments 6 [27,28] and 7 [23,33]. This is because most ISAV sequences reported are of cDNA to mRNA in which the 3' end sequences are removed during polyadenylation of the mRNA transcripts [34]. The sequences missing at the 3' ends of the positive strand for different RNA segments reported in the GenBank Database [32] ranged from 6 to 21 nucleotides (Table 10). In the present study, the 3' sequences of the mRNA transcripts of ADL-ISAV-07 terminated 13-18 nucleotides from the full 3' terminus of cRNA, continuing as a poly(A) tail, which corresponded with the location of the polyadenylation signal (Figure 2). Thus, this is the first report to unambiguously identify the ISAV consensus 3' end sequences (Figure 7). Therefore, the first three nucleotides at the 5' end are 5'-AGU (or 5'-TCA in the cDNA), with the putative polyadenylation signal 13-15 nucleotides downstream of the 5' end terminus of the vRNA. This is also true for other members of *Orthomyxoviridae *[8,15]. Thus the changes detected in the quasispecies may affect the polyadenylation of RNA transcripts of segments 4 and 8 of ISAV strain ADL-ISAV-07. Other features of the ISAV genome, like promoter sequences have not been reported yet. However, by analogy with influenza virus, it is possible to annotate the location of promoters in ISAV. Various studies on influenza virus have reported that the first 12 nucleotides at the 3' end of non-coding regions and the first 13 nucleotides at the 5' end of non-coding regions of all the viral RNA segments form the promoter responsible for replication and transcription [10,55,59,60]. Based on the analogy, it appears that the first 7 nucleotides at 3' end of non-coding region and the first 8 nucleotides at 5' end of non-coding regions of all the ISAV RNA segments may comprise the ISAV promoter (Figure 10). Thus the changes detected in the quasispecies may affect the promoter activity in all the segments of ISAV (ADL-ISAV-07) except segment 5.

**Table 10:** Summary of results of a BLAST search 56 of the GenBank Database 32 for ISAV sequences

Genome segment	ISAV isolate, GenBank Acc. No. and reference of published 5' end	**ISAV isolate, GenBank Acc. No. and reference of published 3' end**^**2**^
**Segment 1**	Bremnes/98, AY168787, [25] [has extra 1 nucleotide]	Bremnes/98, AY168787, [25] [missing terminal 24 nucleotides];
		CCBB, AF404347, [27] [missing terminal 6 nucleotides];
		ADL-PM 3205 ISAV-07, Figure 2, this paper [missing terminal 17 nucleotides]

**Segment 2**	Sotra 92/93, AJ002475, [31] [has extra 12 nucleotides]	Sotra 92/93, AJ002475, [31] [missing terminal 18 nucleotides];
		Bay of Fundy/97, AF262399, Krossoy, Unpublished 1999 [missing terminal 63 nucleotides];
		ADL-PM 3205 ISAV-07, Figure 2, this paper [missing terminal 18 nucleotides]

**Segment 3**	CCBB, AF404345, [27];	CCBB, AF404345, [27] [missing terminal 14 nucleotides];
	NB isolate, AF306549, [26] [has extra 11 nucleotides]	390/98, AJ276858, [57] [missing terminal 14 nucleotides];
		ADL-PM 3205 ISAV-07, Figure 2, this paper [missing terminal 18 nucleotides]

**Segment 4**	NB isolate, AF306548, [26]	CCBB, AF404344, [27] [missing terminal 21 nucleotides];
		SF83/04, AY744391, Plarre and Nylund, Unpublished 2004 [missing terminal 32 nucleotides];
		ADL-PM 3205 ISAV-07, Figure 2, this paper [missing terminal 13 nucleotides]

**Segment 5**	ME/01, AF429986, [27];	ME/01, AF429986, [27];
	CCBB, AF404343, [27];	CCBB, AF404343, [27];
	390/98, AF429988, [27];	390/98, AF429988, [27];
	Norway, AF429987, [27];	Norway, AF429987, [27];
	[all have extra 1 nucleotide]	[all missing terminal 12 nucleotides];
		ADL-PM 3205 ISAV-07, Figure 2, this paper [missing terminal 17 nucleotides]

**Segment 6**	ME/01, AY059402, [27];	CCBB, AF404342, [27];
	CCBB, AF404342, [27];	Glesvaer, AF220607, [28];
	Glesvaer, AF220607, [28];	ADL-PM 3205 ISAV-07, Figure 2, this paper [missing terminal 14 nucleotides]
	Bremnes/98, AF302799, [29]	

**Segment 7**	Glesvaer/2/90^1^, [23];	Glesvaer/2/90^1^, [23];
	NB isolate^1^, [33];	NB isolate, AF328627, [33];
	Bremnes/98, AY044132, [30]	ADL-PM 3205 ISAV-07, Figure 2, this paper [missing terminal 16 nucleotides]

**Segment 8**	Glesvaer/2/90, Y10404, [58] [has extra 9 nucleotides]	Glesvaer/2/90, Y10404, [58] [missing terminal 13 nucleotides];
		NBISA01, AF315063, Kibenge, Unpublished 2000 [missing terminal 18 nucleotides];
		ADL-PM 3205 ISAV-07, Figure 2, this paper [missing terminal 17 nucleotides]

^1^End sequences not in GenBank Database.

^2^Only the longest sequence of each ISAV genotype (European and North American) in the GenBank Database is shown, as all 3' end sequences are incomplete (i.e., do not include 3' terminus) except for genome segments 6 and 7.

It is interesting that in the quasispecies study, all five clones of the 3' NCR of segment 5 cRNA of ADL-ISAV-07 had identical sequence (HI = 0.0), and yet it was the most divergent between the two ISAV genotypes at 77.2% sequence identity. This is even more divergent than at the amino acid level [3]. Conversely, the 3' NCR sequence of segment 3 cRNA of ADL-ISAV-07 had a heterogeneity index of 0.8 and yet it was 100% identical between the two ISAV genotypes. Thus the 5' NCR sequences of segments 1, 2, 3, 5, and 7, and the 3' NCR sequences of segments 3 and 4 cRNA were 100% identical in the two genotypes. These identical sequences were also present in ISAV strains of both genotypes for sequence deposited in the GenBank database [32]. Clearly, the biological significance of ISAV quasispecies warrants further study.

## Conclusions

In conclusion, using sequence analysis of multiple clones derived from one RT-PCR product for each mRNA/cRNA transcript end for the 8 RNA genome segments, we present evidence of intra-segment ISAV quasispecies, with some RNA segments being more prone to genetic changes in their transcripts. The 3' NCR sequence of segment 5 was the least divergent within the viral population but was the most divergent between the two ISAV genotypes. Conversely, the viral population of 3' NCR sequence of segment 3 cRNA transcripts was the most heterogenous but the consensus sequence was identical between the two ISAV genotypes. Moreover we report for the first time the comprehensive unambiguous identification of the 5' and 3' terminal sequences of the 8 RNA genome segments from two strains of ISAV representing the two genotypes of ISAV. Because most ISAV sequences are of cDNA to mRNA, they do not contain the 3' end sequences, which are removed during polyadenylation of the mRNA transcripts. We report for the first time the ISAV consensus sequence CA^T^/_A_TTTTTACT-3' (in the message sense 5'-3') in all segments of both ISAV genotypes.

## Methods

### Viruses

ISAV strains NBISA01 of North American genotype, and ADL-PM 3205 ISAV-07 of the European genotype were used in this study. NBISA01 was propagated in CHSE-214 cell line whereas ADL-PM 3205 ISAV-07 was propagated in the macrophage/dendritic like cell line (TO cell line), and the lysates were harvested as described [61,62].

### RNA extraction

Total RNA was extracted from ISAV-infected TO cells, and vRNA was extracted from purified virus by treating the samples with 1.2 ml of TRIzol reagent (Invitrogen) for 10 minutes, and then adding 300 ul of chloroform followed by centrifugation at 12,000 rpm for 15 minutes. The aqueous phase was loaded on RNeasy Mini Columns and subsequent steps were carried out as per the protocols of RNeasy Mini Kit (Qiagen). The extracted RNA was subsequently treated with DNAse I to remove the contaminating DNA using the RNase-free DNAse I (Qiagen). The purity of RNA was examined using A260/280 ratio based on spectrophotometer readings.

### Amplification of 5' and 3' non-coding sequences of ISAV genome

Attempts were made to use the rapid amplification of cDNA ends (RACE) method to determine the 5' and 3' NCRs of the ISAV genome.

The 5' RACE was performed using a commercially available kit from Invitrogen. In this method, first strand cDNA was synthesized from RNA with M-MLV enzyme (Invitrogen) using the ISAV segment specific primers (ssp1) (Table 2). The synthesized cDNA was purified to remove excess nucleotides and primers. The purified cDNA was polyadenylated with terminal deoxynucleotidyltraferase (TdT) followed by PCR amplification with Abridged Anchor Primer, which is supplied with the 5' RACE kit (Invitrogen), and ISAV segment specific primers (ssp2) (Table 7). The amplified products were gel purified using the Q1A quick gel extraction kit (Qiagen) and were used as templates for performing PCR using the Universal Amplification Primer (UAP) and ISAV segment specific primers (ssp2) (Table 7).

The 3' RACE was performed using a commercially available kit from Invitrogen. In this method, the viral RNA was first polyadenylated by treating the RNA with poly-A-polymerase (Ambion) at 37°C for an hour. The viral RNA was purified using NucAway spin column (Ambion) and used as a template for 3' RACE. The polyadenylated RNA was converted to cDNA using an oligo-dT primer and reverse transcriptase enzyme. The cDNA was then amplified using UAP and either ISAV segment specific primers (ssp3) for total RNA (Table 1) or ISAV segment specific primers (ssp4) for vRNA (Table 6). The amplified products were gel purified using the Q1A quick gel extraction kit (Qiagen) and were cloned in TOPO TA cloning kit (Invitrogen) prior to plasmid DNA sequencing.

### RNA ligation and RT-PCR

The 3' and 5' ends of ISAV RNA were ligated using T4 RNA Ligase (Invitrogen). A duplicate sample of viral RNA was first treated with tobacco acid pyrophosphatase (TAP) (Epicenter) at 37°C for 1 h. It was then purified using NucAway spin column (Ambion) prior to the ligation reaction. RNA (14 ul) was ligated in a total volume of 20 ul in 1 × X T4 RNA Ligase buffer, 40 units of RNase out (Invitrogen) and 40 units of T4 RNA Ligase. The mixture was incubated at 37°C, followed by heat inactivation at 65°C for 15 min. The cDNA was synthesized from circularized RNA using the Superscript III First Strand Synthesis System for RT-PCR (Invitrogen). Subsequently the PCR was carried out in a final volume of 50 ul containing 1X Expand HiFi reaction buffer, 1.5 mM MgCl_2_, 200 uM dNTPs, 2.5 U Expand High Fidelity DNA polymerase (Roche) and forward and reverse ISAV segment specific primers (Tables 3 and 8), each at the concentration of 0.4 uM. The reaction mixture was denatured at 94°C for 2 min followed by 35 cycles of the following conditions: denaturation at 94°C for 30 sec, annealing at 55°C for 30 sec and elongation at 72°C for 1 min. The amplification was completed with one cycle of final elongation at 72°C for 7 min. The amplified products were gel purified using the Q1A quick gel extraction kit (Qiagen).

### Molecular cloning and DNA sequencing

The purified PCR products were cloned in the plasmid pCRII-TOPO using the TOPO TA cloning kit (Invitrogen). The clones were screened by restriction enzyme analysis of plasmid DNA with *Eco*RI and plasmid DNA sequencing was performed on *Eco*RI positive clones by ACGT Corporation (Ontario). The cDNA sequences were deposited in the GenBank Database [32].

### Sequence analysis

Sequences were analysed using the Sequence Manipulation Suite program [63] and the Clustal W program [64].

## Competing interests

KS, NS, and AR are employees of Novartis Animal Health Canada Inc. This does not alter the authors' adherence to all the BioMed Central policies on sharing data and materials.

## Authors' contributions

VK carried out the molecular genetic studies, performed the sequence alignment and drafted the manuscript. MK participated in the original design of the study, prepared the purified virus, and drafting of the manuscript. KS, NS, and AR participated in the original design of the study, and drafting of the manuscript. FK conceived of the study, and participated in its design and coordination, and drafted the manuscript. All authors read and approved the final manuscript.

## References

[B1] Kibenge FS Munir K Kibenge MJ Joseph T Moneke E Infectious salmon anemia virus: causative agent, pathogenesis and immunity Anim Health Res Rev 2004 5 65 78 10.1079/AHR200461 15460541

[B2] Godoy MG Aedo A Kibenge MJ Groman DB Yason CV Grothusen H Lisperguer A Calbucura M Avendaño F Imilán M Jarpa M Kibenge FS First detection, isolation and molecular characterization of infectious salmon anaemia virus associated with clinical disease in farmed Atlantic salmon (Salmo salar) in Chile BMC Vet Res 2008 4 4 28 10.1186/1746-6148-4-28 18680586 PMC2519066

[B3] Kibenge FS Godoy MG Wang Y Kibenge MJ Gherardelli V Mansilla S Lisperger A Jarpa M Larroquete G Avendaño F Lara M Gallardo A Infectious salmon anaemia virus (ISAV) isolated from the ISA disease outbreaks in Chile diverged from ISAV isolates from Norway around 1996 and was disseminated around 2005, based on surface glycoprotein gene sequences Virol J 2009 26 6 88 10.1186/1743-422X-6-88 PMC2710322 19558648

[B4] Johnson A Binette SL Cook-Versloot M Beattie M McGeachy M Gagné N McDonald JT Ritchie RJ Association between ISAV mortalitites and ISAV molecular type in the bay of fundy, Canada Canadian Technical Report of Fisheries Aquatic Sciences 2008

[B5] Dannevig BH Falk K Press CM Propagation of infectious salmon anaemia (ISA) virus in cell culture Vet Res 1995 26 438 442 8581019

[B6] Falk K Namork E Rimstad E Mjaaland S Dannevig BH Characterization of infectious salmon anemia virus, an orthomyxo-like virus isolated from Atlantic salmon (Salmo salar L.) J Virol 1997 71 9016 9023 9371558 10.1128/jvi.71.12.9016-9023.1997 PMC230202

[B7] Kawaoka Y Cox NJ Haller O Hongo S Kaverin N Klenk HD Lamb RA McCauley J Palese P Rimstad E Webster RG Fauquet CM, Mayo MA, Maniloff J, Desselberger U, Ball LA Infectious Salmon Anaemia Virus Virus Taxonomy - Eight Report of the International Committee on Taxonomy Viruses 2005 Elsevier Academic Press: New York 681 693

[B8] de Wit E Bestebroer TM Spronken MI Rimmelzwaan GF Osterhaus AD Fouchier RA Rapid sequencing of the non-coding regions of influenza A virus J Virol Methods 2007 139 85 9 10.1016/j.jviromet.2006.09.015 17059848

[B9] Bouvier NM Palese P The biology of influenza viruses Vaccine 2008 12 26 Suppl 4 D49 53 10.1016/j.vaccine.2008.07.039 PMC3074182 19230160

[B10] Wang L Lee CW Sequencing and mutational analysis of the non-coding regions of influenza A virus Vet Microbiol 2009 135 239 47 10.1016/j.vetmic.2008.09.067 18986781

[B11] Desselberger U Racaniello VR Zazra JJ Palese P The 3' and 5'-terminal sequences of influenza A, B, and C virus RNA segments are highly concerved and show partial inverted complementarity Gene 1980 8 315 328 10.1016/0378-1119(80)90007-4 7358274

[B12] Robertson JS 5' and 3' terminal nucleotide sequences of the RNA genome segments of influenza virus Nucleic Acids Res 1979 6 3745 3757 10.1093/nar/6.12.3745 493121 PMC327975

[B13] Skehel JJ Hay AJ Nucleotide sequences at the 5' termini of influenza virus RNAs and their transcripts Nucleic Acids Res 5 1207 1219 10.1093/nar/5.4.1207 652519 PMC342071

[B14] Hsu MT Parvin JD Gupta S Krystal M Palese P Genomic RNAs of influenza viruses are held in a circular conformation in virions and in infected cells by a terminal panhandle Proc Natl Acad Sci USA 1987 84 8140 8144 10.1073/pnas.84.22.8140 2446318 PMC299494

[B15] Li X Palese P Characterization of the polyadenylation signal of influenza virus RNA J Virol 1994 68 1245 9 7507179 10.1128/jvi.68.2.1245-1249.1994 PMC236570

[B16] Hagen M Chung TD Butcher JA Krystal M Recombinant influenza virus polymerase: requirement of both 5' and 3' viral ends for endonuclease activity J Virol 68 1509 1515 8107213 10.1128/jvi.68.3.1509-1515.1994 PMC236607

[B17] Lee MT Klumpp K Digard P Tiley L Activation of influenza virus RNA polymerase by the 5' and 3' terminal duplex of genomic RNA Nucleic Acids Res 2003 31 1624 1632 10.1093/nar/gkg253 12626703 PMC152857

[B18] Fodor E Pritlove DC Brownlee GG Characterization of the RNA-fork model of Virion RNA in the initiation of transcription in influenza A virus J Virol 1995 69 4012 4019 7769659 10.1128/jvi.69.7.4012-4019.1995 PMC189134

[B19] Luyjtes W Krystal M Enami M Parvin JD Palese P Amplification, expression, and packaging of foreign gene by influenza virus Cell 1989 59 1107 1113 10.1016/0092-8674(89)90766-6 2598262

[B20] Tchatalbachev S Flick R Hobom G The packaging signal of influenza viral RNA molecules RNA 2001 7 979 989 10.1017/S1355838201002424 11453070 PMC1370150

[B21] Zheng H Palese P Garcia-Sastre A Nonconserved nucleotides at the 3' and 5' ends of an influenza A virus RNA play an important role in viral RNA replication Virology 1996 217 242 251 10.1006/viro.1996.0111 8599209

[B22] Jackson D Cadman A Zurcher T Barclay WS A reverse genetics approach for recovery of recombinant influenza B viruses entirely from cDNA J Virol 2002 76 11744 7 10.1128/JVI.76.22.11744-11747.2002 12388735 PMC136801

[B23] Sandvik T Rimstad E Mjaaland S The viral RNA 3'- and 5'-end structure and mRNA transcription of infectious salmon anaemia virus resemble those of influenza viruses Arch Virol 2000 145 1659 69 10.1007/s007050070082 11003475

[B24] Toennessen R Lauscher A Rimstad E Comparative aspects of infectious salmon anaemia virus, an orthomyxovirus of fish, to influenza viruses Indian J Microbiol 2009 49 308 314 10.1007/s12088-009-0055-4 PMC3450196 23100790

[B25] Snow M Ritchie R Arnaud O Villoing S Aspehaug V Cunningham CO Isolation and characterisation of segment 1 of the infectious salmon anaemia virus genome Virus Res 2003 92 99 105 10.1016/S0168-1702(02)00322-2 12606081

[B26] Ritchie RJ Heppell J Cook M Griffiths S Identification and characterization of segments 3 and 4 of the ISAV genome Virus Genes 2001 22 289 297 10.1023/A:1011110105819 11450947

[B27] Clouthier SC Rector T Brown NEC Anderson ED Genomic organization of infectious salmon anaemia virus J Gen Virol 2002 83 421 428 11807235 10.1099/0022-1317-83-2-421

[B28] Rimstad E Mjaaland S Snow M Mikalsen AB Cunningham CO Characterization of the infectious salmon anaemia virus genomic segment that encodes the putative haemagglutinin J Virol 2001 75 5352 5356 10.1128/JVI.75.11.5352-5356.2001 11333916 PMC114940

[B29] Krossøy B Devold M Sanders L Knappskog PM Aspehaug V Falk K Nylund A Koumans S Endresen C Biering E Cloning and identification of the infectious salmon anaemia virus haemagglutinin J Gen Virol 2001 82 1757 1765 11413388 10.1099/0022-1317-82-7-1757

[B30] Biering E Falk K Hoel E Thevarajan J Joerink M Nylund A Endresen C Krossøy B Segment 8 encodes a structural protein of infectious salmon anaemia virus (ISAV); the co-linear transcript from Segment 7 probably encodes a non-structural or minor structural protein Dis Aquat Organ 2002 49 117 122 10.3354/dao049117 12078979

[B31] Krossøy B Hordvik I Nilsen F Nylund A Endresen C The putative polymerase sequence of infectious salmon anaemia virus suggests a new genus within the *Orthomyxoviridae* J Virol 1999 73 2136 2142 9971796 10.1128/jvi.73.3.2136-2142.1999 PMC104458

[B32] GenBank Database http://www.ncbi.nlm.nih.gov/genbank/

[B33] Ritchie RJ Bardiot A Melville K Griffiths S Cunningham CO Snow M Identification and characterization of the genomic segment 7 of the infectious salmon anaemia virus genome Virus Res 2002 84 161 170 10.1016/S0168-1702(01)00375-6 11900848

[B34] Lamb RA Choppin PW Gene structure and replication of influenza virus Annu Rev Biochem 1983 52 467 506 10.1146/annurev.bi.52.070183.002343 6351727

[B35] Kibenge FSB Kibenge MJ Wang Y Qian B Hariharan S McGeachy S Mapping of putative virulence motifs on infectious salmon anaemia virus surface glycoprotein genes J Gen Virol 2007 88 3100 3111 10.1099/vir.0.83097-0 17947536

[B36] Nylund A Plarre H Karlsen M Fridell F Otten KF Baratland A Sæther PA Transmission of infectious salmon anaemia virus in farmed populations of Atlantic salmon (Salmo salar) Arch Virol 2007 152 151 179 10.1007/s00705-006-0825-9 16941061

[B37] Gagné N Ritchie RJ Viral nomenclature: Toward standardisation Annual Meeting of the Fish Health Section of the American Fisheries Society 2008 University of Prince Edward Island, Charlottetown, PEI, Canada http://ocs.vre.upei.ca/index.php/FHS/FHS2008/paper/view/212

[B38] Lyngstad TM Jansen PA Sindre H Jonassen CM Hjortaas MJ Johnsen S Brun E Epidemiological investigation of infectious salmon anaemia (ISA) outbreaks in Norway 2003-2005 Prev Vet Med 2008 84 213 227 10.1016/j.prevetmed.2007.12.008 18243376

[B39] Stech J Xiong X Scholtissek C Webster RG Independence of evolutionary and mutational rates after transmission of avian influenza viruses to swine J Virol 1999 73 1878 84 9971766 10.1128/jvi.73.3.1878-1884.1999 PMC104428

[B40] Domingo E Baranowski E Ruiz-Jarabo CM Martin-Hernandez AM Saiz JC Quasispecies structure and persistence of RNA viruses Emerg Infect Dis 1998 4 521 527 10.3201/eid0404.980402 9866728 PMC2640251

[B41] Eigen M On the nature of virus quasispecies Trends Microbiol 1996 4 216 8 10.1016/0966-842X(96)20011-3 8795155

[B42] Kongchanagul A Suptawiwat O Kanrai P Uiprasertkul M Puthavathana P Auewarakul P Positive selection at the receptor-binding site of haemagglutinin H5 in viral sequences derived from human tissues J Gen Virol 2008 89 1805 10 10.1099/vir.0.2008/002469-0 18632950

[B43] Ramakrishnan MA Tu ZJ Singh S Chockalingam AK Gramer MR Wang P Goyal SM Yang M Halvorson DA Sreevatsan S The feasibility of using high resolution genome sequencing of influenza A viruses to detect mixed infections and quasispecies PLoS One 2009 4 9 e7105 10.1371/journal.pone.0007105 19771155 PMC2740821

[B44] Holland JJ De La Torre JC Steinhauer DA RNA virus populations as quasispecies Curr Top Microbiol Immunol 1992 176 1 20 1600747 10.1007/978-3-642-77011-1_1

[B45] Domingo E Holland JJ RNA virus mutations and fitness for survival Annu Rev Microbiol 1997 51 151 178 10.1146/annurev.micro.51.1.151 9343347

[B46] Wang S Liu Q Pu J Li Y Keleta L Hu YW Liu J Brown EG Simplified recombinational approach for influenza A virus reverse genetics J Virol Methods 2008 151 74 78 10.1016/j.jviromet.2008.03.020 18456344

[B47] Sampath R Russell KL Massire C Eshoo MW Harpin V Blyn LB Melton R Ivy C Pennella T Li F Levene H Hall TA Libby B Fan N Walcott DJ Ranken R Pear M Schink A Gutierrez J Drader J Moore D Metzgar D Addington L Rothman R Gaydos CA Yang S St George K Fuschino ME Dean AB Stallknecht DE Goekjian G Yingst S Monteville M Saad MD Whitehouse CA Baldwin C Rudnick KH Hofstadler SA Lemon SM Ecker DJ Global surveillance of emerging Influenza virus genotypes by mass spectrometry PLoS One 2007 2 5 e489 10.1371/journal.pone.0000489 17534439 PMC1876795

[B48] Plotch SJ Bouloy M Ulmanen I Krug RM A unique cap(m7GpppXm)-dependent influenza Virion endonuclease cleaves capped RNAs to generate the primers that initiate viral RNA transcription Cell 1981 23 847 858 10.1016/0092-8674(81)90449-9 6261960

[B49] Szymkowiak C Kwan WS Su Q Toner TJ Shaw AR Youil R Rapid method for the characterization of 3' and 5' UTRs of influenza viruses J Virol Methods 2003 107 15 20 10.1016/S0166-0934(02)00184-2 12445933

[B50] Mathet VL López JL Ruiz V Sánchez DO Carballal G Campos RH Oubiña JR Dynamics of a hepatitis B virus e antigen minus population ascribed to genotype F during the course of a chronic infection despite the presence of anti-HBs antibodies Virus Res 2007 123 72 85 10.1016/j.virusres.2006.08.004 16979773

[B51] Baez M Zazra JJ Elliott RM Young JF Palese P Nucleotide sequence of the influenza A/duck/Alberta/60/76 virus NS RNA: conservation of the NS1/NS2 overlapping gene structure in a divergent influenza virus RNA segment Virology 1981 113 397 402 10.1016/0042-6822(81)90166-5 6927848

[B52] Fuller TL Saatchi SS Curd EE Toffelmier E Thomassen HA Buermann W Desante DF Nott MP Saracco JF Ralph CJ Alexander JD Pollinger JP Smith TB Mapping the risk of avian influenza in wild birds in the US BMC Infect Dis 2010 10 1 187 10.1186/1471-2334-10-187 20573228 PMC2912310

[B53] Nakada S Graves PN Palese P The influenza C virus NS gene: evidence for a spliced mRNA and a second NS gene product (NS2 protein) Virus Res 1986 4 263 73 10.1016/0168-1702(86)90005-5 2943090

[B54] Weber F Haller O Kochs G Conserved vRNA end sequences of Thogoto-orthomyxovirus suggest a new panhandle structure Arch Virol 1997 142 1029 33 10.1007/s007050050138 9191867

[B55] Fodor E Pritlove DC Brownlee GG The influenza virus panhandle is involved in the inititation of transcription Virol 1994 68 4092 4096 10.1128/jvi.68.6.4092-4096.1994 PMC236924 8189550

[B56] Altschul SF Gish W Miller W Myers EW Lipman DJ Basic local alignment search tool J Mol Biol 1990 215 403 410 2231712 10.1016/S0022-2836(05)80360-2

[B57] Snow M Cunningham CO Characterisation of the putative nucleoprotein gene of infectious salmon anaemia virus (ISAV) Virus Res 2001 74 111 118 10.1016/S0168-1702(00)00248-3 11226579

[B58] Mjaaland S Rimstad E Falk K Dannevig BH Genomic characterization of the virus causing infectious salmon anemia in Atlantic salmon (Salmo salar L.): an orthomyxo-like virus in a teleost J Virol 1997 71 7681 6 9311851 10.1128/jvi.71.10.7681-7686.1997 PMC192118

[B59] Jackson D Elderfield RA Barclay WS Molecular studies of influenza B virus in the reverse genetics era J Gen Virol 2010 10.1099/vir.0.026187-0 20926635

[B60] Crow M Deng T Addley M Brownlee GG Mutational analysis of the influenza virus cRNA promoter and identification of nucleotides critical for replication J Virol 2004 78 6263 70 10.1128/JVI.78.12.6263-6270.2004 15163719 PMC416531

[B61] Kibenge FS Lyaku JR Rainnie D Hammell KL Growth of infectious salmon anaemia virus in CHSE-214 cells and evidence for phenotypic differences between virus strains J Gen Virol 2000 81 143 50 10640552 10.1099/0022-1317-81-1-143

[B62] Kibenge FS Gárate ON Johnson G Arriagada R Kibenge MJ Wadowska D Isolation and identification of infectious salmon anaemia virus (ISAV) from Coho salmon in Chile Dis Aquat Organ 2001 45 9 18 10.3354/dao045009 11411649

[B63] Sequence Manipulation Suite program http://www.ualberta.ca/~stothard/javascript/index.html

[B64] Clustal W program http://www.ebi.ac.uk/Tools/clustalw2/index.html

